# VETCONNECT: A VA TELEHEALTH PROGRAM PROVIDING TREATMENT, EDUCATION AND TRANSITIONS OF CARE TO NURSING HOME VETERANS

**DOI:** 10.1093/geroni/igz038.1228

**Published:** 2019-11-08

**Authors:** Cari Levy, Anne Hale, Emily Galenbeck, Leah M Haverhals

**Affiliations:** 1 VA Rocky Mountain Regional VA Medical Center, Aurora, Colorado, United States; 3 Seattle-Denver Center of Innovation, Denver, Colorado, United States

## Abstract

Transporting nursing home Veterans to hospitals for outpatient care can present many challenges, including lengthy time in transit, coordination difficulties between the hospital and nursing home, and travel burden on Veterans. In June 2017, the VetConnect program began offering Veterans in Colorado residing in Department of Veterans Affairs (VA) contracted Community Nursing Homes (CNHs) and State Veteran Nursing Homes (SVHs) telehealth appointments in Geriatrics, Mental Health, Palliative Care, and Psychiatry via clinicians at the Rocky Mountain Regional VA Medical Center (VAMC). Then in June 2018, VetConnect expanded to offer Virtual Tours for Veterans transitioning out of inpatient care to CNHs the opportunity to virtually tour the CNH, connecting with CNH staff through video for a more stress-free and informed transition from hospital to CNH. In winter 2018, VetConnect expanded once more to include Dementia Educational Outreach Series for CNH and SVH staff, allowing them to connect virtually to VA staff with expertise in dementia care and other Veteran-specific topics such as treating post-traumatic stress disorder (PTSD). To date, VetConnect has provided 502 telehealth visits, 18 Virtual Tours, and 4 dementia ECHO trainings (with 58 nursing home staff participants). Evaluation measures to be presented include collection and analysis of telehealth nurse field notes (who are present with Veterans at CNHs/SVHs during telehealth visits), creation of process maps, conducting key stakeholder interviews, and identification of potential impacts the VetConnect program has had on VA to CNH and SVH relationships and Veteran health.

